# Study on Mechanical Properties and Weakening Mechanism of Acid Corrosion Lamprophyre

**DOI:** 10.3390/ma15196634

**Published:** 2022-09-24

**Authors:** Jun Guo, Xincheng Mi, Guorui Feng, Tingye Qi, Jinwen Bai, Xiaoze Wen, Ruipeng Qian, Linjun Zhu, Xingchen Guo, Luyang Yu

**Affiliations:** 1 College of Mining Technology, Taiyuan University of Technology, Taiyuan 030024, China; 2 Shanxi Province Coal-Based Resources Green and High-Efficiency Development Engineering Center, Taiyuan 030024, China; 3 Shanxi Coking Coal Group Co., Ltd., Taiyuan 030024, China

**Keywords:** acid corrosion, lamprophyre, mechanical activation behavior, weakening mechanism

## Abstract

In order to study the weakening mechanism and mechanical behaviors of hard lamprophyre of Carboniferous Permian coal-bearing strata in China’s mining area, lamprophyre samples were subjected to static rock dissolution experiments with pH values of 0, 2, and 4. The acid corrosion mechanism of lamprophyre was revealed from the weight changes of samples, characteristics of solution ion concentration, and macro-mechanical properties. The experimental results show that reaction occurred between lamprophyre and acid solution. With the increasing concentration of H^+^, the reaction was more intense, the degree of acid etching was higher, and the weight loss was greater. The internal damage induced by acid etching results in the slow extension of the compaction stage of stress–strain curve of uniaxial compression, and the obvious deterioration of mechanical properties of the lamprophyre. The uniaxial compressive strength of the lamprophyre in the dry state is 132 MPa, which decreased to 39 MPa under the acid etching condition, showing significant mudding characteristics. Dolomite (CaMg(CO_3_)_2_ with 19.63%) and orthoclase (KAlSi_3_O_8_ with 31.4%) in lamprophyre are the major minerals constituents involved in acidification reaction. Photomicrograph recorded from SEM studies reveals that the dissolution effect was directly related to the concentration of H^+^ in the solution. The dissolution effect was from the surface to the inside. The small dissolution pores became larger and continuously expanded, then finally formed a skeleton structure dominated by quartz. The content of K^+^, Ca^2+^, and Mg^2+^ in the solution after acid etching reaction indicates that the acidified product of orthoclase is colloidal H_2_SiO_3_, which adhered to the surface of samples during acid etching and hinders the further acidification of minerals. The dissolution of dolomite and orthoclase under acidic conditions directly leads to the damage of their structure and further promotes the water–rock interaction, which is the fundamental reason for the weakening of the mechanical properties of lamprophyre.

## 1. Introduction

Hard igneous rock formed by magmatic intrusion in many coal-bearing sedimentary strata in China seriously restrict safe and efficient mining of coal resources. Using a case study of the Datong mining area as an example, the intrusive range of lamprophyre is 30.5 km^2^ in Carboniferous Permian No. 3–5 extra thick coal seam of the mining area. Igneous rocks occur at the top of coal seam in the form of layered rock dykes. From east to west, the thickness of rock dykes decreases from 8 m to 0 m [1]. The main mineral compositions of lamprophyre are orthoclase (31.4%), dolomite (19.63%), quartz (9.23%), and clay minerals [2]. The uniaxial compressive strength of the lamprophyre is 132 MPa in the dry state. The intrusion of lamprophyre causes the accumulation of elastic strain energy of hard roof in the process of stress redistribution during the mining on No. 3–5 coal seam, which will cause risks such as strong strata behavior and may increase the risk of rock burst and sudden roof collapse [3]. At the same time, it induces difficulties in advancing the working face of No. 4 coal seam [4]. In addition, the erosion of igneous rock destroys the structure of coal and rock mass, bringing more challenges to supporting the stability of mines [5]. The hard property of the lamprophyre is the main factor affecting the mining of the coal seam in the invaded area—whether the lamprophyre is intruded into the coal seam or into the roof of the coal seam. Therefore, the rupture and softening of the hard rock strata are important means to understand that the above risks factors and the weakening of its mechanical properties are the fundamental causes for solving the mining engineering problems.

The weakening effect of traditional hydraulic fracturing is limited in the stress environment, with large thickness, dense texture, and high stress on the hard rock. Acidification is more likely to cause the change of mineral composition and structure at the micro-fracture of rock mass to achieve weakening effect [6]. Fracturing acidification technology has become an effective method to solve the problem of hard rock fracture mechanism [7]. Therefore, studying the weakening mechanism by acidic chemical solution is important for the development of engineering structures. Huo, Li et al. [8,9,10] studied the degradation of mechanical properties of sandstone and the effect of acid corrosion under variable conditions and suggested that the concentration of cations such as K^+^, Ca^2+^, and Mg^2+^ in acid solution played an important role in understanding the degradation factor of mechanical properties in rock samples. Yao et al. [11] pointed out that the corrosion of Na_2_SO_4_ aqueous solution had different effects on the structure and composition of artificially developed fractured limestone. Feng et al. [12,13,14,15] studied the mechanical properties and failure characteristics of sandstone, granite, and limestone to understand the effects of chemical corrosion, and they analyzed the microscopic fracture behavior of rock which underwent chemical corrosion. He, Guo et al. [16] studied the influence mechanism of acid solution by making a comparison among the mechanical properties of limestone which had undergone the static corrosion of gelled acid and viscosifying acid. Next, they concluded that the difference of acid solution on the internal corrosion structure of rock core was the fundamental reason for the difference of mechanical strength. Liu et al. [17] studied the strength degradation of argillaceous sandstone after acid dry–wet cycles and pointed out that acid environment had an obvious degradation effect on shear strength of sandstone. Feng, Wang et al. [18,19,20,21] studied the mechanical properties of sandstone under hydrochemical action and pointed out that the water–rock interaction tends to be stable with the extension of soaking time under the limited hydrochemical environment. Liu, Cui et al. [22,23,24] studied the microscopic characteristics of carbonate rock acid etching and clarified the characteristics changes of microscopic pore structure in carbonate rock before and after acid etching. Yang, Xue et al. [25] established igneous rock mechanics parameters based on logging data inversion. However, there are relatively few studies on the physical, chemical, mechanical properties, and acid etching mechanism of lamprophyre after acid solution corrosion [26].

In this paper, the failure of mechanical properties of acid-etched lamprophyre is studied. The mechanical properties of lamprophyre undergoing static corrosion by acid solution with different pH values are studied by a uniaxial loading mechanical test. Based on the changes of cation concentration and pH value in acid solution, the acid-etch corrosions of lamprophyre are explored, which provides fundamental guidance for the acid-etch deterioration of rock mass, confronting the exploitation of coal resource in the area of igneous rock intrusion.

## 2. Materials and Methods

### 2.1. Preparation of Samples

Rock samples in this test were taken from the lamprophyre intrusive layer of the Permian coal measure strata in Datong mining area. The sampling and sample preparation were strictly carried out in accordance with the coal industry standard of the People’s Republic of China GB 235615-2009-T. Among these standard samples of φ50 mm × 100 mm, the samples without cracks and surface defects were selected, while the abnormal wave velocity samples were eliminated after ultrasonic testing.

### 2.2. Test Procedures

The lamprophyre rocks of Carboniferous Permian coal measures strata in Datong mining area were strongly carbonated. This rock is mainly composed of carbonated mica, orthoclase, quartz, and other minerals. It is identified as carbonated quartz-bearing monzodiorite based on the identification results of Taiyuan Mineral Resources Supervision and Inspection Center of Ministry of Land and Resources. It is also called lamprophyre in Tashan mining area. Dolomite (CaMg(CO_3_)_2_) and orthoclase (KAISi_3_O_8_), covering over 50%, are the main minerals of lamprophyre. Besides, a certain amount of quartz and clay minerals (chlorite, kaolinite, illite, and montmorillonite) [27] are also contained. The detailed mineral composition of lamprophyre is shown in Table 1.

**Figure 1:**
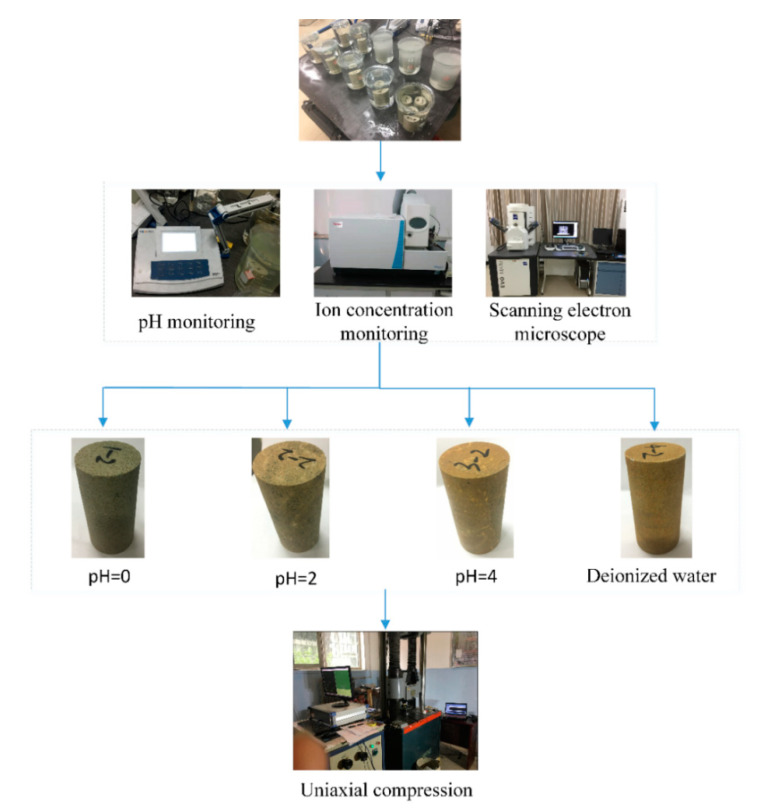
Acidizing tests on lamprophyre.

Dolomite and orthoclase efficiently react with acid solutions, which promotes significant change in chemical composition. Therefore, it is feasible to soften lamprophyre by acidification. In order to further quantitatively study the acidizing effect of H^+^ concentration of acid solution on lamprophyre and explore the acid etching mechanism of lamprophyre, a static corrosion test of lamprophyre was designed, as shown in Figure 1.

Twelve samples, after processing and screening, were selected for the present study and divided into four groups. These four groups of samples were immersed in four different types of solutions (deionized water, pH = 0, pH = 2, pH = 4) of 2000 mL for the static acid etching experiment conducted for 480 h at room temperature (about 25 °C). During the static acid etching, the pH value of each solution and the weight of the samples were continuously measured by pH meter and electronic balance when the concentration of some cations in the immersion solution was monitored. In addition, the mechanical properties and failure characteristics of lamprophyre under uniaxial loading were studied [28,29]. Based on the above data, the acidification mechanism of lamprophyre was analyzed.

## 3. Mechanical Properties Analysis

### 3.1. Stress–Strain Relation

**Figure 2:**
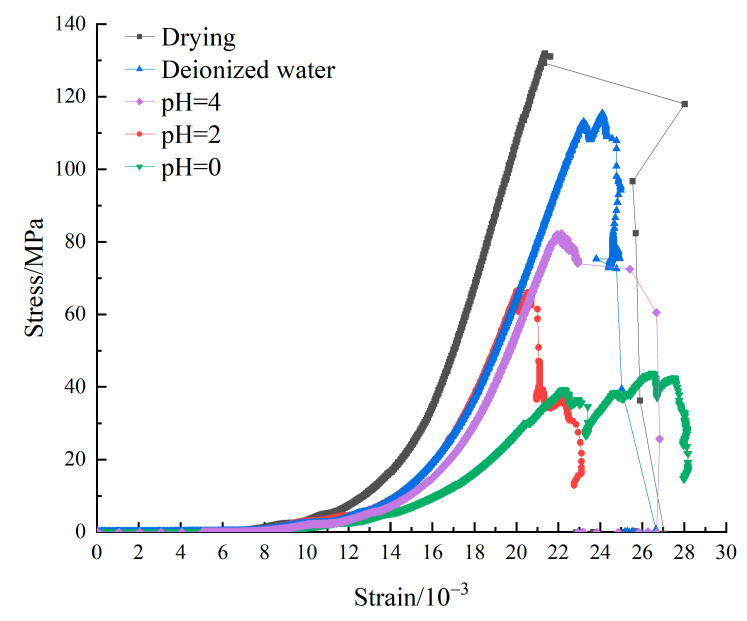
Stress–strain curves of lamprophyre under uniaxial compression.

The stress–strain curves of different samples under uniaxial loading were shown in Figure 2. Table 2 shows the average peak stress and strength damage of samples of each group. The dried samples were procured by drying samples in the oven under the condition of 105 °C for 24 h. Samples of each group show the typical compaction—elasticity—plastic yield—post-peak four stages of change characteristics. The post-peak curve stage was gradually completed with the decrease of pH value. However, the compaction stage and yield stage of the stress–strain curve gradually slowed down and extended with the decrease of pH value. The total strain value of the compaction stage of the sample immersed in pH = 0 solution was 1.5 times that of the dry sample. The ultimate strain of the whole sample also showed an increasing trend. It indicated that the increase of acid etching holes and cracks in the sample changed the microstructure of the lamprophyre. At the same time, the properties of grains were changed. Acid etching softened the lamprophyre sample and increased its plastic deformation.

The fluctuation before the stress–strain curve is decreased and generally considered to be caused by internal damage and the shear slip of weak surface. It can be seen from Figure 2 that the increase of strain and the brittle characteristics of the samples in the drying group samples were observed. Each group of samples after acid etching has significant development of shear slip section. The shear slip section strain of the samples in the pH = 0 group accounted for 20% of the total strain. It can be seen that a large number of internal damage and weak surfaces appeared in the lamprophyre samples after acid etching. Due to the increase of pH value of acid solution, the pre-peak accumulation deformation energy decreased significantly (the samples with pH = 0 were about 1/3 of the dry samples). The change in structure and grain size of samples after acid etching led to the sudden decrease of elastic strain energy accumulation ability of the lamprophyre samples. The post-peak loss deformation energy gradually decreased (about 1/5). It indicated that the damage dissipation energy and the damage severity decreased.

It can be seen from Table 2 and Figure 2 that the strength of the deionized water treatment group was 21 MPa lower than that of the dry control group, and the peak stress damage caused by the dissolution of minerals for cementation such as clays in the lamprophyre was 15.91%. Under the same conditions, the peak stress reduction effect of static acid etching samples was more obvious, and the peak stress damage increased with the decrease of solution pH. The peak stress of the samples soaked in pH = 0 solution mostly decreased. Its peak stress of uniaxial compression is only 39 MPa. It was only 35.13% of the samples soaked in deionized water, and 70.45% lower than that of the samples of the drying group.

**Figure 3:**
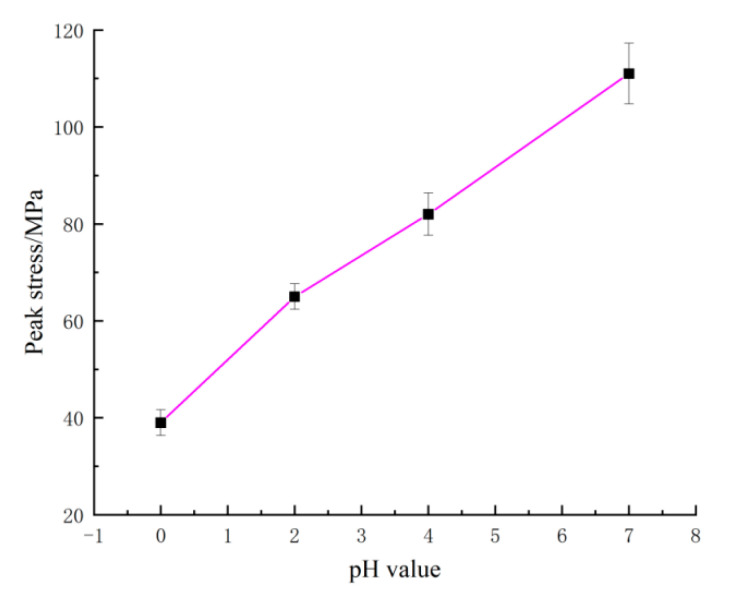
Peak stress–pH value curves of lamprophyre.

As shown in Figure 3, the compressive strength of samples decreased linearly with the decrease of pH value, and the degradation effect of acid solution on the lamprophyre increased linearly with the increase of H^+^ concentration. It was the result of the weakened of mechanical properties caused by internal damage. Due to the increase of H^+^ concentration, the corrosion effect on lamprophyre samples increased, the mass loss rate increased significantly, the internal damage increased, and the mechanical properties deteriorated prominently. The concentration of metal cations in the solution increased significantly.

### 3.2. Weight Changes of Samples

**Figure 4:**
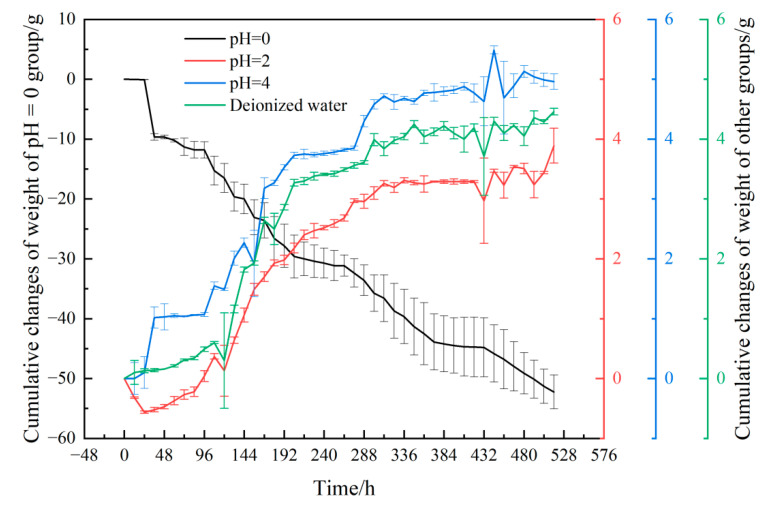
Changes of weight of lamprophyre samples in different solutions.

Acid leaching of lamprophyre is an intensive process of diffusion controlled by water absorption of rock and chemical reaction between rock and acid solution. It is a kind of simultaneous binary process, coupled with physical and chemical actions on rock samples. These effects can be reflected by testing the weight changes of samples. The cumulative variation of weight of four groups of lamprophyre samples in the test are shown in Figure 4. In addition to pH = 0, the increased stage of the cumulative change value of weight of other three groups all showed the rule of exponential function. Then, the cumulative variation of weight became a fixed value with the saturation of water absorption and the end of the chemical reaction. Finally, the weight of the three groups increased compared with the beginning of the test. The pH = 2 group increased by 3.5 g and the pH = 4 group increased by 4.8 g. The weight of samples of deionized water control group only increased under the physical effect of water absorption.

In the solution of pH = 2, the weight of samples decreased in the first 24 h in the dominant stage of chemical reaction. Due to the decrease of H^+^ concentration and the consumption of reactive minerals on the surface of samples, the H^+^ penetration path was prolonged, and the chemical reaction rate was reduced. At the same time, the corrosion of the surface of samples increased the contact area between samples and water, resulting in the acceleration of the absorption of water and the gradual increase of the weight of samples. However, affected by the internal chemical reaction, the increase of the weight of samples in this group was minimum.

In the solution of pH = 4, the water absorption rate was higher than the chemical reaction that causes corrosion and weight loss rate. The weight of the sample increased throughout the test. Affected by the chemical reaction corrosion holes, the final water absorption was higher than that of the deionized water control group.

In the solution of pH = 0, the chemical reaction degree of the lamprophyre sample was intense. The weight was basically linearly reduced. Finally, the weight loss of the sample was more than 50 g.

The above laws showed that the lamprophyre samples showed obvious aboriginal mineral dissolution under the acidification of strong acid solution, which causes an increase in the porosity inside the samples. Compared with the deionized water control group, the weight growth of the sample in pH = 2 solution was less than that in pH = 4 solution, which indicated that acidification destroyed the structural composition of the sample and further promoted the water–rock interaction.

### 3.3. Failure Characteristics of Uniaxial Compression

**Figure 5:**
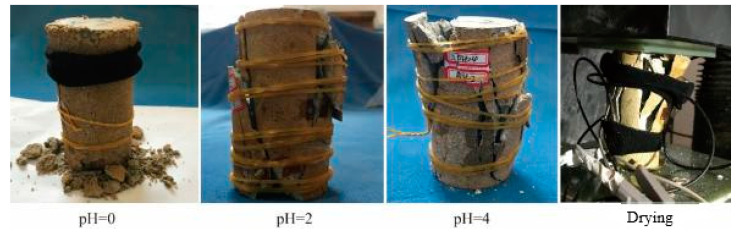
Failure features of lamprophyre.

The failure characteristics are presented as the final evolution state of internal microcracks. The failure characteristics contain rich information, such as sample deformation and force transmission path [30,31,32,33]. Therefore, it is of great significance to analyze them. The lamprophyre rock was a typical hard brittle material in the experiment, but the acid etching effect weakened its brittle characteristics significantly. As shown in Figure 5, the failure characteristics of the sample immersed in pH = 0 solution were consistent with the typical plastic failure. It was in the state of argillization [34]. The debris spalling occurred at both ends of the sample at low stress. Finally, the main cracks were generated along the central axis of the sample, leading to its loss of bearing capacity.

Under the influence of a large number of acid etching holes on the surface and the action of low axial load, severe plastic failure and particle spalling first occurred at both ends of the sample. Before the overall instability, the deformation had not been transferred to the central strain gauge position, and the strain in the middle position did not show obvious change rules during the failure.

The failure of samples in the pH = 2 and pH = 4 solution immersion groups showed typical shear failure characteristics. When pH = 2, shear spalling and failure instability along the conical surface of the sample, and the central part of the sample was relatively complete. When pH = 4, the surface of the sample was flaked off, and the whole sample was broken into a large number of small pieces along axial cracks.

The samples in the drying control group had plate splitting failure. The samples were broken into several pieces of plates along the axial direction. Rock burst, surface cracking, and block ejection occurred during the loading process. The samples were highly broken.

According to the failure characteristics and strain variation of samples, it can be found that the radial strains of different samples are similar. However, the axial strain is significantly different. For the sample immersed in pH = 2 solution, the maximum axial strain was not more than 1600. The axial strain of the sample immersed in pH = 4 solution was close to 2500. The axial strain of the dry sample reached 3500. It indicated that a large amount of elastic strain energy was accumulated in the loading and unloading process of dry lamprophyre samples, which is also the root cause of rockburst when it is damaged [35]. However, the accumulation ability of elastic strain energy of the sample decreased significantly. The samples changed from brittle failure to elastic–plastic failure after acidification. The failure mode of the samples showed a weakening of the fracture degree.

## 4. Characteristics and Mechanism of Acid Erosion

### 4.1. Reaction Process and Phenomena

Carbonate in lamprophyre reacts with the acid solution as follows:

(1) $${2H}^{+}{+\mathrm{CO}}_{3}^{2-}{=\mathrm{CO}}_{2}↑{+H}_{2}O$$

Carbonate minerals participating in the reaction were dissolved. In addition, the dissolution of clay minerals causes some insoluble particles that broke into small pieces to fall off, which floated in the solution and eventually formed precipitation with insoluble products of the reaction while the generated CO_2_ was escaping.

**Figure 6:**
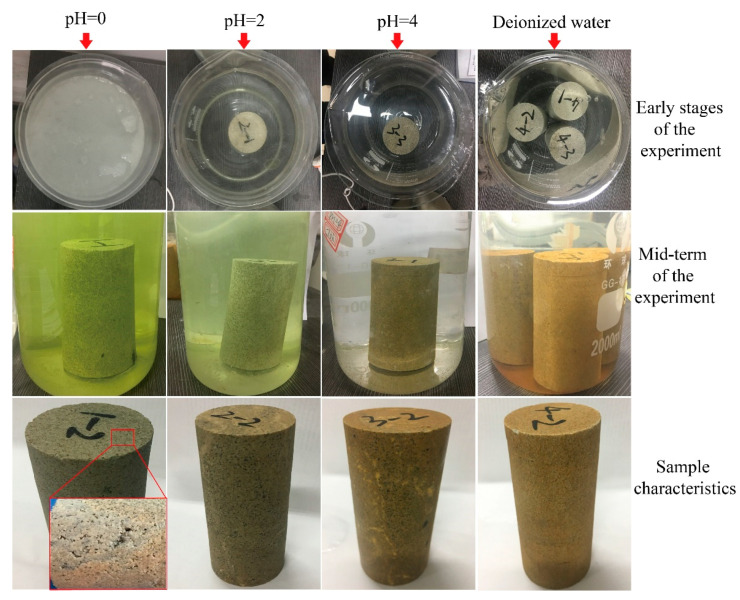
Phenomenon of lamprophyre samples in pickling experiment.

The lamprophyre samples were immersed in acid solutions with different H^+^ concentrations. At the beginning of the experiment, the diffusion of acid solution in the samples played a leading role. Then, fierce chemical reactions occurred on the surface of the sample in the strong acid solution. As shown in Figure 6, severe chemical reactions occurred in the strong acid solution with pH = 0. A large number of bubbles were generated on the sample and a large number of lamprophyre particles were carried during the escape of bubbles. The solution was in a state of emulsion-white turbidity as a whole. The intensity of reaction in pH = 2 solution was lower than that in pH = 0 solution. There were bubbles on the surface of the sample and white particles suspended in the solution. In pH = 4 solution, only a small number of bubbles attached to the surface of the sample were observed, and the solution remained clear. There was no visible change in the samples in deionized water. Therefore, it can be indicated that lamprophyre can react strongly with acid solution—the higher the H^+^ concentration was, the more intense the chemical reaction on the lamprophyre rock samples would be.

With the increase of immersion time, the infiltration path of acid solution into the sample was prolonged. Thus, the intensity of chemical reaction in strong acid solution at this stage was weakened to some extent. The diffusion of acid solution was hindered by the cement produced by pre-reaction and the water film during gas escape. In this process, the chemical action of acid solution plays a leading role. However, the dominant roles of diffusion and chemical action constantly switched when a certain part inside the samples was focused.

When pH = 0, there was still a large number of bubbles on the samples. It indicated that there was still a continuous chemical reaction. Suspended particles were still visible in the solution and the color of the solution changed from milky white to light yellow. A thick layer of insoluble particle precipitation layer was visible at the bottom of the beaker. In the solution of pH = 2, there were bubbles occasionally escaping from the sample, and a small number of white particles suspended in the solution. The color of the solution after etching became light green, and a thin precipitation layer covered the bottom of the beaker. In the solution of pH = 4, the color change was not observed. There was a small number of insoluble particles at the bottom. Due to water absorption of the samples in deionized water, the color of the solution changed from initial grayish white to light yellow. The precipitation at the bottom of the beaker and the color change of the solution shows that the higher the solution H^+^ concentration was, the greater the overall mass losses of samples caused by dissolution of mineral components would be.

The samples of each group were taken out and compared after the test. As shown in Figure 6, in the solution of pH = 0, a large number of corrosion holes with different depths could be seen on the surface of the sample. Some holes were connected into pieces. The appearance of the samples was similar to that of the porous rock. The surface of the samples was affected by the corrosion reaction of minerals such as dolomite and orthoclase. The color of the surface changed from grayish white to grayish black. When pH = 2, the corrosion holes could also be seen on the surface of the samples. However, the number and size of the holes were decreased as compared with pH = 0 conditions. The color of the samples changed to gray-black. When pH = 4, the smoothness of the surface of the samples decreased. The color of local surface of the samples became gray-black. In deionized water, the color of the surface changed from gray to light yellow due to water absorption.

### 4.2. The Changes of pH Value

**Figure 7:**
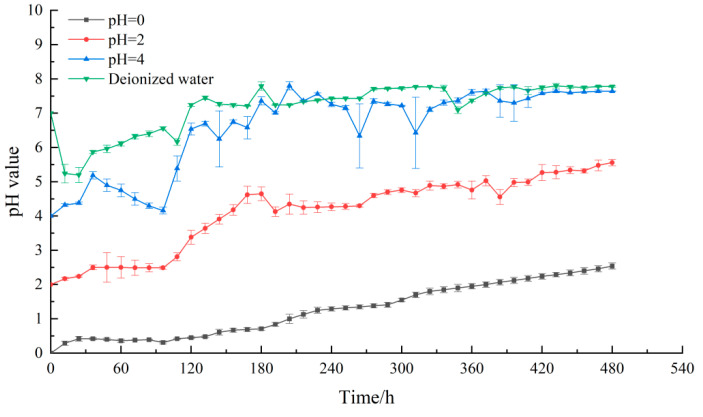
pH changes of the different solutions in tests.

The change of pH value is a spontaneous response to the change of solution H^+^ concentration [36,37,38]. Its monitoring results can reflect the acidification process of lamprophyre well. The monitoring results of pH value of each solution are shown in Figure 7. The pH values of the four solutions showed an increasing trend until they gradually became neutral. The decrease of pH in the early stage of the deionized water group may be caused by the hydrolysis of strong acid and weak alkali salts in minerals. In the two solutions with high H^+^ concentration, the acidification of lamprophyre was observed. At the beginning of the test, there was a period of rapid pH growth which then entered a stable and slow period towards the end. As the reaction continued, the pH change curve fluctuated and increased, and the slope of the curve did not decrease significantly. It can also be seen that in the reaction process of the samples, the dominant position of diffusion and chemical action was continuously switching. The structural damage caused by chemical reaction and the water–rock interaction caused by diffusion promoted each other and jointly affected the acid corrosion process of lamprophyre. The pH value of the solution of pH = 0 and pH = 2 did not reach 7, which indicated that the acidification reaction was still slow. The deionized solution and pH = 4 solution eventually tend to neutral, which can be considered as an indicator of the end of acidification.

### 4.3. Microstructure Characteristics of Scanning Electron Microscopy

**Figure 8:**
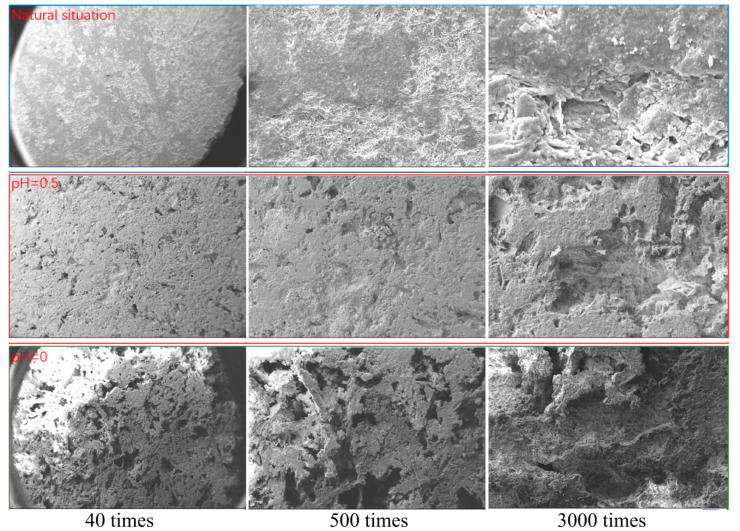
Microstructure characteristics of samples under different acid etching conditions.

The microstructure characteristics of lamprophyre samples under different concentrations of acid solution corrosion were obtained by EVO MA 10 Zeiss scanning electron microscope. The results were shown in Figure 8. The lamprophyre samples were relatively dense under natural conditions. When the magnification is 40 times, the surface of the samples was flat and dense, and basically no voids can be seen. When the magnification is 500 and 3000 times, it is found that there are small pores in the local area of the samples due to the difference of mineral composition.

Observed corrosion cavity appeared on the surface of the sample after the lamprophyre samples were eroded by the solution of pH = 0.5. It was more obvious that the corrosion effect had been carried out to a certain depth inside the sample when the magnification was 500 times. When pH = 0, compared with the solution erosion effect of pH = 0.5, the corrosion degree of the sample was more serious. Almost all the surface of the sample was corroded with holes. The uncorroded part was quartz mineral with a content of 9.32%. The corrosion depth and range of internal minerals were significantly expanded, and the damage degree of the sample was serious when the magnification was 3000 times. It indicated that the dissolution effect was directly related to the concentration of H^+^ in the solution. The higher the concentration was, the greater the degree of dissolution on the lamprophyre samples would be. The dissolution effect on the samples was from the surface to the inside. The dissolution pores became larger and continue to expand. Finally, the skeleton structure, most of which was quartz minerals, was formed.

### 4.4. Variation Analysis of K^+^, Ca^2+^ and Mg^2+^ Concentrations

The mineral composition analysis of lamprophyre shows that the minerals involved in acid solution reaction are orthoclase and dolomite. The chemical reaction equations are [39]:

(2) $${\mathrm{KAlSi}}_{3}O_{8}{+4H}^{+}{+H}_{2}{O=K}^{+}{+\mathrm{Al}}^{3+}{+3H}_{2}{\mathrm{SiO}}_{3}\left(\mathrm{colloid}\right)$$

(3) $${\mathrm{CaMg}(\mathrm{CO}}_{3}{)}_{2}{+4H}^{+}{=\mathrm{Ca}}^{2+}{+\mathrm{Mg}}^{2+}{+2\mathrm{CO}}_{2}↑{+2H}_{2}O$$

**Figure 9:**
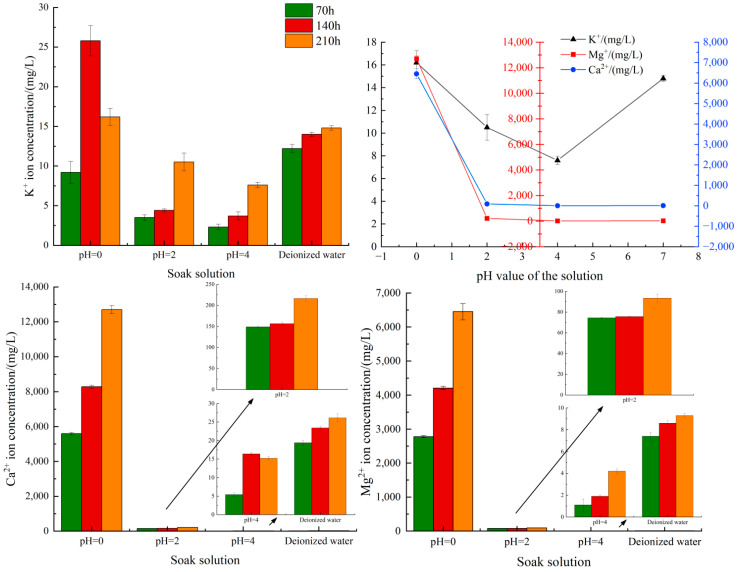
Concentrations of K^+^, Ca^2+^, and Mg^2+^ in different solutions.

Therefore, the acidification process of lamprophyre can be monitored by measuring the concentration variation of K^+^, Ca^2+^ and Mg^2+^ in solution at different time periods. In this regard, four kinds of solutions were sampled at 70 h, 140 h, and 210 h, respectively. The concentrations of three ions in the solution were determined by inductively coupled plasma emission spectrometer. The results are shown in Table 3 and Figure 9.

It was found that the concentrations of K^+^, Ca^2+^, and Mg^2+^ in each solution increased with the increase of reaction time. At the end of the experiment, the ion concentration decreased with the increase of pH of the solution. The ion concentration in the solution at pH = 0 was significantly higher than that in the rest groups. The concentrations of Ca^2+^ and Mg^2+^ were 2~3 orders of magnitude higher than those in the rest groups. It indicated that the acidizing dissolution of lamprophyre was positively correlated with H^+^ concentration. Dolomite played a leading role in the acidizing reaction of lamprophyre under strong acid conditions. Mineral acid etching directly leads to structural damage and further promotes water–rock interaction, which is the fundamental cause of weakening in mechanical properties of lamprophyre.

In the experiment, it was found that flocculent colloid attached to the surface of the sample. Based on the chemical reaction equation of orthoclase and dolomite with acid solution, it can be told that the colloid, generated by the reaction of orthoclase with H^+^, was H_2_SiO_3_. The H_2_SiO_3_ colloid was directly adsorbed on the surface of the reactant, blocking the continuous contact reaction between KAlSi_3_O_8_ and H^+^. The concentration of K^+^ was two orders of magnitude lower than that of Ca^2+^ and Mg^2+^ in the ion concentration measured at 210 h. It further confirmed the effect of H_2_SiO_3_ colloid on rock acid etching. This was also the reason why dolomite with 19.63% content in lamprophyre was the main mineral involved in acidification rather than the orthoclase with higher content. This conclusion enlightens us that the way to prevent the formation of H_2_SiO_3_ colloid in the acidification reaction of syenite is important to further improve the acidification effect when using acidification to reduce the fracturing pressure of hard rock strata of lamprophyre.

In addition, the determination results at 210 h showed that the concentration of Ca^2+^ in the acid solution at pH = 0 reached 1300 mg/L, and the concentration of Mg^2+^ was 6500 mg/L. The difference in the concentration of Ca^2+^ and Mg^2+^ indicated that in the samples of lamprophyre, other minerals containing Ca^2+^ might be involved in the acidification reaction.

### 4.5. Strength Weakening Mechanism

**Figure 10:**
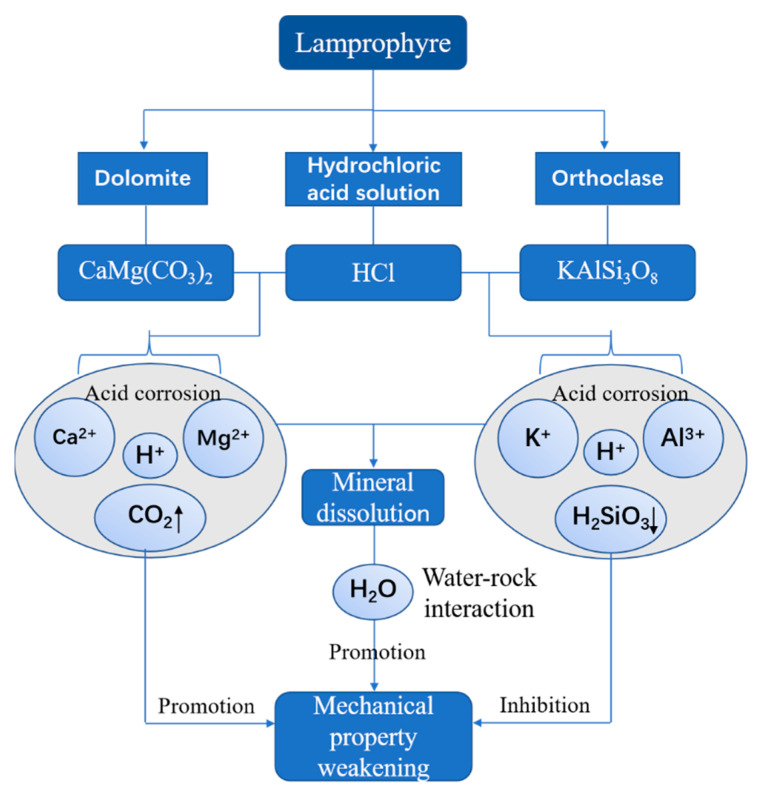
Weakening mechanism and mechanical behaviors of acid corrosion lamprophyre.

Dolomite and orthoclase are the main minerals of lamprophyre. They are also the main minerals directly involved in the acidification reaction. As shown in Figure 10, strong acidification occurs when dolomite encounters H^+^ and rapidly dissolves into Ca^2+^ and Mg^2+^. At the same time, CO_2_ bubbles are generated. The generation of bubbles accelerates the dissolution of ions, further promoting the acidification reaction and the dissolution of minerals. Meanwhile, orthoclase dissolves into K^+^ and Al^3+^ under the action of H^+^, which thus leads to the dissolution of lamprophyre. Mineral dissolution caused by acidification increases the contact area between mineral aggregates and solution and is beneficial to the weakening of mechanical properties of lamprophyre samples caused by acidification and water–rock interaction. However, with the progress of acidification, colloidal H_2_SiO_3_ is gradually formed on the mineral surface and attached to the lamprophyre surface. With the increase of the adhesive area of H_2_SiO_3_ colloid, the samples are gradually isolated from the solution to inhibit the acidification reaction. It can be seen that the acid etching process of lamprophyre can be divided into three stages. The first stage is the strong acidification reaction stage accompanied by bubbles, which is the main stage of corrosion damage. Secondly, there is the relatively stable stage of the interaction between acid etching and water–rock. Finally, there is the stage of corrosion weakening, which is caused by the adhesion of H_2_SiO_3_ colloid.

This paper only concludes that the production of H_2_SiO_3_ colloid in the acidification reaction hinders the further development of acid etching. How the influence of H_2_SiO_3_ colloid can be reduced on the acidification effect is still worth studying.

## 5. Conclusions

(1) Lamprophyre and acid solution easily generate a strong acid salt reaction. When the concentration of H^+^ is higher, there would be an increase in the intensity of the reaction, the degree of acid etching, the dissolution rate, and the weight loss rate of minerals caused by acid etching.

(2) With the decrease of solution pH, the acid etching effect significantly weakens the brittle characteristics of lamprophyre, making it gradually argillization. The accumulation ability of pre-peak elastic strain energy decreases significantly, and the severity of damage decreases significantly during the loading process.

(3) The peak stress decreases linearly with the decrease of pH value, and the maximum strength damage can reach 70% after acid etching of lamprophyre. The dissolution of dolomite under acidic condition directly leads to structural damage. It further promotes water–rock interaction, which is the fundamental reason for the weakening of mechanical properties of lamprophyre.

(4) The acid etching process of lamprophyre can be divided into three stages: the strong acidification reaction stage, the relatively stable stage of acid etching and water–rock interaction, and the corrosion weakening stage caused by the adhesion of H_2_SiO_3_ colloid. When using acidification to reduce the fracturing pressure of hard rock strata of lamprophyre, how to prevent orthoclase from producing H_2_SiO_3_ colloid in acidification is important for the further improvement of acidification effect.

## Figures and Tables

**Table 1:** Mineral composition and content of lamprophyre.

Mineral Component	Dolomite	Orthoclase	Chlorite	Loweite	Montmorillonite	Illite	Pyrophyllite	Quartz	Pyroxmangite
Content/%	19.63	31.4	1.46	13.16	3.11	1.67	6.70	9.32	13.64

**Table 2:** Peak stress of different lamprophyre under uniaxial compression.

	Peak Stress/MPa	Peak Stress Damage/%
Drying	132 ± 4	0
Deionized water	111 ± 7	15.91
pH = 4 solution	82 ± 5	37.88
pH = 2 solution	65 ± 3	50.76
pH = 0 solution	39 ± 3	70.45

**Table 3:** Concentrations of K^+^, Na^+^, Ca^2+^, and Mg^2+^ in different solutions.

Soaking Solution	K^+^ Ion Concentration (mg/L)	Ca^2+^ Ion Concentration (mg/L)	Mg^2+^ Ion Concentration (mg/L)
Deionized water (70 h)	12.2 ± 0.6	19.4 ± 0.7	7.4 ± 0.4
Deionized water (140 h)	14.0 ± 0.4	23.4 ± 0.5	8.6 ± 0.3
Deionized water (210 h)	14.8 ± 0.3	26.1 ± 1.2	9.3 ± 0.2
pH = 0 (70 h)	9.2 ± 1.6	5594.4 ± 81.7	2778.2 ± 34.2
pH = 0 (140 h)	25.8 ± 2.2	8290.3 ± 96.3	4209.1 ± 56.1
pH = 0 (210 h)	16.2 ± 1.2	12,700.2 ± 245.3	6451.9 ± 275.3
pH = 2 (70 h)	3.5 ± 0.4	148.8 ± 2.1	74.4 ± 0.8
pH = 2 (140 h)	4.4 ± 0.2	156.4 ± 3.6	75.6 ± 0.3
pH = 2 (210 h)	10.5 ± 1.3	216.3 ± 7.3	93.4 ± 4.6
pH = 4 (70 h)	2.3 ± 0.4	5.4 ± 0.5	1.1 ± 0.6
pH = 4 (140 h)	3.7 ± 0.6	16.4 ± 0.4	1.9 ± 0.1
pH = 4 (210 h)	7.6 ± 0.4	15.2 ± 0.5	4.2 ± 0.2

## Data Availability

Not applicable.

## References

[1] Guo J., Feng G.R. (2018). Roof Strata Behavior and Support Resistance Determination for Ultra-Thick Longwall Top Coal Caving Panel: A Case Study of the Tashan Coal Mine. Energies.

[2] Guo J., Feng G.R. (2018). Dynamic Mechanical Behavior of Dry and Water Saturated Igneous Rock with Acoustic Emission Monitoring. Shock. Vib..

[3] Feng G.R., Sun Q., Guo J. (2019). Numerical Simulation Study on Effect of Lamprophyre in Water on Overburden Migration in Ultrathick Coal Seam. Saf. Coal Mines.

[4] Yang Z.W. (2013). Research on the distribution law of the mining supporting pressure 4^#^ coal seam in Yongdingzhuang Mine of Datong Mine Zone. China Coal.

[5] Guo J.G. (2017). Study on control of surrounding rock of tunnel with very thick coal seam intruded by igneous rock. J. Henan Polytech. Univ. Nat. Sci..

[6] Ling S.X., Wu X.Y., Sun C.W., Liao X., Ren Y., Li X.N. (2016). Experimental Study of Chemical Damage and Mechanical Deterioration of Black Shale Due Water-Rock Chemical Action. J. Exp. Mech..

[7] Deng G.Z., Liu H. (2021). Fracture mechanism of acid fracturing of sandstones gangue layer in fully mechanized mining face. Saf. Coal Mines.

[8] Huo R.K., Han F., Li S.G., Wang G.J. (2019). Experimental study on physicochemical and mechanical properties of acid-corroded sandstone. J. Xi’an Univ. Archit. Technol. Nat. Sci. Ed..

[9] Shu G.L., Huo R.K. (2018). Experimental Study on Physicomechanical Properties of Sandstone under Acidic Environment. Adv. Civ. Eng..

[10] Huo R.K., Li S.G. (2018). CT Analysis on Mesoscopic Structure of Sandstone under Acidic Environment. Indian J. Geo-Mar. Sci..

[11] Yao H.Y., Feng X.T., Cui Q., Shen L.F., Zhou H., Cheng C.B. (2009). Experimental study of effect of chemical corrosion on strength and deformation of hard brittle limestone. Rock Soil Mech..

[12] Ding W.X., Feng X.T. (2004). Testing Study on Mechanical Effect for Limestone under chemical Erosion. Chin. J. Rock Mech. Eng..

[13] Chen S.L., Feng X.T., Li S.J. (2003). The fracturing behaviors of Three Gorges granite under chemical erosion. Rock Soil Mech..

[14] Feng X.T., Wang C.Y. (2002). Testing Study and Real-Time Observation of Rock Meso-Cracking Process under Chemical Erosion. Chin. J. Rock Mech. Eng..

[15] Chen S.L., Feng X.T. (2002). The effect of chemical erosion on mechanical behaviors of Xiaolangdi sandstone. Rock Soil Mech..

[16] He C.M., Guo J.C. (2013). Mechanism Study of Acid on Mechanical Properties of Limestone. Chin. J. Rock Mech. Eng..

[17] Liu X.R., Li D.L., Wang Z., Zhang L. (2016). The effect of dry-wet cycles with acidic wetting fluid on strength deterioration of shaly sandstone. Chin. J. Rock Mech. Eng..

[18] Deng H.F., Zhi Y.Y. (2019). Mechanical properties of sandstone and damage evolution of microstructure under water-rock interact. Rock Soil Mech..

[19] Feng X.W., Wang W. (2018). A rheological damage model of sandstone under water-rock chemical interaction. Rock Soil Mech..

[20] Wang W., Li X.H. (2017). Experimental study of mechanical characteristics of sandy slate under chemical corrosion. Rock Soil Mech..

[21] Yu J., Zhang X. (2019). Meso-damage and mechanical properties degradation of sandstone under combined effect of water chemical corrosion and freeze-thaw cycles. Rock Soil Mech..

[22] Liu H.B., Cui S. (2020). Study on the Microstructure and Mechanical Properties of Carbonate Rock before and after Acidification. Chin. J. Undergr. Space Eng..

[23] Cui B., Feng P.Y. (2021). Study on Microscopic Characteristics of Carbonate Acid Corrosion of M Oilfield in Middle East. J. Guangdong Univ. Petrochem. Technol..

[24] Li X.Y., Wu H.C. (2021). Influences of Microstructural Differences on Acid Corrosive Damage to Carbonate Rocks. Xinjiang Pet. Geol..

[25] Yang H., Xue X.J. (2021). Establishment of igneous rock mechanical parameters model based on electric logging data inversion and its engineering application. Nat. Gas Ind..

[26] Shen C., Feng G.R. (2017). The Influence of and Mechanism Study Tensile Mechanical Properties of Lamprophyre in Acid Condition. J. Taiyuan Univ. Technol..

[27] Guo J., Feng G.R. (2015). Mechanical property variation under dynamic uniaxial compression and micro-mechanism of lamprophyre in saturated state. J. China Coal Soc..

[28] Chen W., Wu L. (2022). Research on Uniaxial Compression Mechanics of Diorite under Flowing Acidic Solution Scouring. Minerals.

[29] Luo X.Y., Cao P. (2022). Mechanical Behaviour of Anchored Rock Containing Weak Interlayer under Uniaxial Compression: Laboratory Test and Coupled DEM–FEM Simulation. Minerals.

[30] Zhang P.F., Zhang Y.B. (2019). Experimental Research on Deformation Characteristics of Waste-Rock Material in Underground Backfill Mining. Minerals.

[31] Liu G.S., Li L. (2017). An Investigation of the Uniaxial Compressive Strength of a Cemented Hydraulic Backfill Made of Alluvial Sand. Minerals.

[32] Liu T.Y., Cui M.Y. (2022). Fracture and Damage Evolution of Multiple-Fractured Rock-like Material Subjected to Compression. Materials.

[33] Wu S., Qin G.P. (2022). Deformation, Failure, and Acoustic Emission Characteristics under Different Lithological Confining Pressures. Materials.

[34] Qi G.Q., Huang R.Q. (2004). Evolution of Mineral under Interaction of Water and Crystalline Rock. Mineral. Petrol..

[35] Liu Z.X., Wang W. (2020). Method of energy evolution of rock under uniaxial compression test. J. China Coal Soc..

[36] Xue J.C., Dong L.J. (2022). Effect of Chemical Corrosion and Axial Compression on the Dynamic Strength Degradation Characteristics of White Sandstone under Cyclic Impact. Minerals.

[37] Yu H.J., Liu H.L. (2022). Deformation and Failure Mechanism of Weakly Cemented Mudstone under Tri-Axial Compression: From Laboratory Tests to Numerical Simulation. Minerals.

[38] Chen L., Jia B.X. (2022). Study on Mechanical Behavior and Energy Mechanism of Sandstone under Chemical Corrosion. Materials.

[39] Luo X.J., Yang W.D. (2001). Effects of pH on the Solubility of the Feldspar and the Development of Secondary Porosity. Bull. Mineral. Petrol. Geochem..

